# Complete genome sequences of 14 lytic bacteriophages against *Acinetobacter baumannii* from Kenya belonging to the genera *Obolenskvirus* and *Friunavirus*

**DOI:** 10.1128/mra.01193-24

**Published:** 2025-02-25

**Authors:** Felix Mwai, Collins Kigen, Celestine Makobe, Ivy Mutai, Martin Georges, Erick Odoyo, Moses Gachoya, Lillian Musila

**Affiliations:** 1 Centre for Microbiology Research, Kenya Medical Research Institute 118982 , Nairobi, Kenya; 2 School of Biomedical Sciences, College of Health Sciences, Jomo Kenyatta University of Agriculture and Technology, Nairobi, Kenya; 3 Microbiology Hub, Walter Reed Army Institute of Research- Africa, Kericho, Kenya; 4 Kenya Institute of Primate Research, Nairobi, Kenya; 5 School of Veterinary Medicine and Science, University Of Nottingham, Nottingham, England, United Kingdom; Portland State University, Portland, Oregon, USA

**Keywords:** *Acinetobacter*, bacteriophages, Kenya, multidrug resistance, phage therapy, *Obolenskvirus*, *Friunavirus*

## Abstract

We report the genome sequences of 14 *Acinetobacter* phages isolated in Kenya, belonging to the genera *Friunavirus* and *Obolenskvirus*. They have double-stranded DNA with genome sizes between 39 and 45 kb and G + C content from 38% to 39%. The genomes contain 56 to 93 predicted coding sequences.

## ANNOUNCEMENT

*Acinetobacter baumannii* is a World Health Organization priority pathogen that causes nosocomial and drug-resistant infections (1, 2). There is, therefore, an urgent need for an alternative mechanism for the treatment of multidrug-resistant (MDR) *A. baumannii* infections, with bacteriophages (phages) offering a promising solution (3–5). Fourteen phages targeting clinical MDR *A. baumannii* isolates were isolated from water samples collected from domestic effluent, streams, dams, drainage points in residential areas, informal settlements, and sewage treatment plants within Nairobi County, Kenya.

The water samples were transported and stored at 4°C prior to processing. Phages were isolated and enriched using clinical isolates of MDR *A. baumannii* as hosts (IRB approval #WRAIR2089/KEMRI2767), as shown in Table 1, using the direct spot test method, as described by Soro *et al*., 2024 (6). A well-isolated phage plaque on tryptic soy agar (TSA) was picked and resuspended in 500 µL of sodium chloride–magnesium sulfate buffer, incubated (4°C, 24 hours) to allow phages to dissociate from the agar, filtered (0.22 µm), and plaque assays performed for phage purification by three rounds of single-plaque isolation using the *A. baumannii* enrichment strains. Pure phages were amplified on the enrichment strain in 50 mL tryptic soy broth (TSB) and concentrated by centrifugation (10,000 × *g*, 18 hours) to reach a titer of >10^10^ PFU/mL for DNA extraction (7). Bacterial host RNA and DNA were removed with DNAse and RNAse, respectively, and phage DNA extracted using the Norgen phage DNA isolation kit (Norgen Biotek Corp., Canada), as described by Soro *et al*., 2024 (6). Sequencing libraries were then prepared using the NEBNext (New England Biolabs, Massachusetts – USA) and native barcoding kit (SQK-NDB114) and loaded onto a MinION FLO-MIN114 R10.4.1 flowcell (Oxford Nanopore Technologies, Oxford Science Park, United Kingdom) and sequenced on a Nanopore sequencer, which yielded single-end reads with an N50 of 10.98 kb. Dorado v0.7.0 was used for basecalling, trimming adapters, and filtering off reads with Qscore <8. The raw reads were assessed for quality with Nanoplot v.14.2 (8) and assembled using Flye 2.9.4 (9). Pharokka v1.7.1 (10) was used to annotate the genomes, determine the taxonomy against the INPHARED database (11), and screen for antimicrobial resistance (AMR) and virulence genes against the Comprehensive Antibiotic Resistance Database (CARD) (12) and Virulence Factors database (VFDB) (13), respectively. PhageLeads (14) was used to screen for lysogenic genes and predict the lifecycle. CheckV v1.0.3 (15) was used to assess genome completeness. Default parameters were used for all software tools. Phage termini could not be determined due to incompatibility between the long reads and the existing bioinformatic tools. All phages had double-stranded DNA, and their genomic sizes ranged between 39,867 and 45,389 bp with a GC% ranging from 38% to 39% and contained 56–93 predicted coding sequences (Table 1). The phage families and genera results are presented in Table 1. There are 110 *Friunavirus* and 60 *Obolenskvirus* phage genomes in the NCBI Nucleotide Database with 92%–99% identity to our study genomes. All the phage genomes lacked AMR, virulence, and lysogeny genes, suggesting a strictly virulent lifecycle and are promising candidates for therapeutic and environmental control applications.

**TABLE 1:** Genomic characteristics of the *A. baumannii* phages

Phage name	Sample collection source	Year of phage isolation	GPS coordinate	Isolation/enrichment strain	Family	Genus	Genome length (bp)	GC content (%)	No. of CDS	Genome coverage (x)	Genome completeness (%)	No. of raw reads	Read N50	GenBank accession No.	SRA accession no.
Acinetobacter phage vB_Ab_1137_KEN_01	Domestic effluent	2022	*1°19'05.3"S 36°48'23.7"E*	*A. baumannii ST 1137*	*Autographiviridae*	*Friunavirus*	40,684	39	63	896	98.22	39375	7891	PP841126	SRR28834052
Acinetobacter phage vB_Ab_01_KEN_01	Urban stream	2022	*1°19'05.3"S 36°48'23.7"E*	*A. baumannii ST 1*	*Unclassified*	*Obolenskvirus*	43,518	38	92	883	96.71	14057	14879	PP841127	SRR28834051
Acinetobacter phage vB_Ab_02_KEN_01	Dam wastewater	2022	*1°19'05.3"S 36°48'23.7"E*	*A. baumannii ST 2*	*Autographiviridae*	*Friunavirus*	41,229	39	61	373	99.53	14178	15053	PP841128	SRR28834060
Acinetobacter phage vB_Ab_01_KEN_02	Dam wastewater	2022	*1°19'05.3"S 36°48'23.7"E*	*A. baumannii ST 1*	*Unclassified*	*Obolenskvirus*	43,522	38	92	1657	96.72	24260	14786	PP841129	SRR28834059
Acinetobacter phage vB_Ab_1137_KEN_02	Urban stream	2022	*1°19'05.3"S 36°48'23.7"E*	*A. baumannii ST 1137*	*Autographiviridae*	*Friunavirus*	39,867	39	62	890	96.24	60686	7927	PP841130	SRR28834058
Acinetobacter phage vB_Ab_1137_KEN_03	Urban stream	2022	*1°17'13.2"S 36°50'40.5"E*	*A. baumannii ST 1137*	*Autographiviridae*	*Friunavirus*	42,412	39	65	396	100	13281	14225	PP841131	SRR28834057
Acinetobacter phage vB_Ab_164_KEN_01	Urban stream	2022	*1°17'13.2"S 36°50'40.5"E*	*A. baumannii ST 164*	*Autographiviridae*	*Friunavirus*	40,649	39	59	547	98.12	18166	13272	PP841132	SRR28834056
Acinetobacter phage vB_Ab_164_KEN_02	Urban stream	2022	*1°17'13.2"S 36°50'40.5"E*	*A. baumannii ST 164*	*Autographiviridae*	*Friunavirus*	41,204	39	59	83	99.46	11646	11280	PP841133	SRR28834055
Acinetobacter phage vB_Ab_02_KEN_02	Sewage from treatment plant	2022	*1°14'37.1"S 37°01'51.3"E*	*A. baumannii ST 2*	*Unclassified*	*Obolenskvirus*	45,068	38	92	846	100	25051	14192	PP841134	SRR28834054
Acinetobacter phage vB_Ab_02_KEN_03	Sewage from treatment plant	2022	*1°14'00.7"S 37°01'32.3"E*	*A. baumannii ST 2*	*Unclassified*	*Obolenskvirus*	45,389	38	93	1175	100	13015	15134	PP841135	SRR28834053
Acinetobacter phage vB_Ab_1137_KEN_04	Sewage from treatment plant	2022	*1°14'37.1"S 37°01'51.3"E*	*A. baumannii ST 1137*	*Autographiviridae*	*Friunavirus*	41,115	39	66	371	99.26	15620	12694	PP841136	SRR28834050
Acinetobacter phage vB_Ab_1137_KEN_05	Domestic effluent	2022	*1°16'59.4"S 36°41'27.2"E*	*A. baumannii ST 1137*	*Autographiviridae*	*Friunavirus*	40,153	39	56	125	96.93	7380	12381	PP841137	SRR28834049
Acinetobacter phage vB_Ab_1137_KEN_06	Domestic effluent	2022	*1°16'59.4"S 36°41'27.2"E*	*A. baumannii ST 1137*	*Autographiviridae*	*Friunavirus*	41,054	39	60	234	99.11	15696	11893	PP841138	SRR28834048
Acinetobacter phage vB_Ab_164_KEN_03	Domestic effluent	2022	*1°17'13.2"S 36°50'40.5"E*	*A. baumannii ST 164*	*Autographiviridae*	*Friunavirus*	41,168	39	60	187	99.38	10166	16631	PP841139	SRR28834061

## Data Availability

The *Acinetobacter* phage genome sequences are available in NCBI under the BioProject number PRJNA1104989. The individual GenBank and Sequence Read Archive (SRA) accession numbers are listed in Table 1.
